# A Flexible Alternative to the Cox Proportional Hazards Model for Assessing the Prognostic Accuracy of Hospice Patient Survival

**DOI:** 10.1371/journal.pone.0047804

**Published:** 2012-10-17

**Authors:** Branko Miladinovic, Ambuj Kumar, Rahul Mhaskar, Sehwan Kim, Ronald Schonwetter, Benjamin Djulbegovic

**Affiliations:** 1 Center for Evidence Based Medicine and Health Outcomes Research, University of South Florida, Tampa, Florida, United States of America; 2 HPC Healthcare, Temple Terrace, Florida, United States of America; 3 H. Lee Moffitt Cancer Center & Research Institute, Tampa, Florida, United States of America; Memorial Sloan Kettering Cancer Center, United States of America

## Abstract

Prognostic models are often used to estimate the length of patient survival. The Cox proportional hazards model has traditionally been applied to assess the accuracy of prognostic models. However, it may be suboptimal due to the inflexibility to model the baseline survival function and when the proportional hazards assumption is violated. The aim of this study was to use internal validation to compare the predictive power of a flexible Royston-Parmar family of survival functions with the Cox proportional hazards model. We applied the Palliative Performance Scale on a dataset of 590 hospice patients at the time of hospice admission. The retrospective data were obtained from the Lifepath Hospice and Palliative Care center in Hillsborough County, Florida, USA. The criteria used to evaluate and compare the models' predictive performance were the explained variation statistic R^2^, scaled Brier score, and the discrimination slope. The explained variation statistic demonstrated that overall the Royston-Parmar family of survival functions provided a better fit (R^2^ = 0.298; 95% CI: 0.236–0.358) than the Cox model (R^2^ = 0.156; 95% CI: 0.111–0.203). The scaled Brier scores and discrimination slopes were consistently higher under the Royston-Parmar model. Researchers involved in prognosticating patient survival are encouraged to consider the Royston-Parmar model as an alternative to Cox.

## Introduction

Prognostic models are often used to estimate the length of patient survival and improvement in the accuracy of prognosis translates into superior quality of patient care. Precise prognosis of survival using modeling techniques requires rigorous methods for the development and testing of the accuracy of prognostic models. Developing a prognostic model entails having accurate patient data for prognosis, and selecting clinically relevant candidate predictors and measures of model performance, usually in the context of multivariable regression [1]. This process produces patient performance scores that allow for classification of patients into different risk groups [2], [3], [4].

In the hospice setting, accurate prognostication of survival affords patients and their families a vital opportunity to attend to matters such as planning, prioritizing, and preparing for death [5]. Predicting patient survival is a complex decision making process involving numerous subjective and numerical factors that have substantial variation which may lead to poor prediction of life expectancy. Many physicians practice optimism or avoidance, thus overestimating survival at times by a factor of five [6]. Implementing appropriate statistical methodologies translates into improved accuracy of prognosis and superior quality of care. Predictions based on appropriate statistical modeling have been shown to be superior to physicians' prognostication [4], [7].

The Cox proportional hazards (CPH) model [8] is the most commonly-used survival prediction model [4], [9]. In the hospice and palliative settings, demographic and clinical covariates are often included in CPH to predict patient survival [10], [11]. The appeal of the model is its analytic simplicity and that the baseline survival function does not need to be defined *apriori*–it is absorbed when the likelihood function is maximized (note that “baseline” refers to zero values of the covariates, not to time equal to zero). It is possible to estimate the baseline survival function for the CPH model conditional on the estimated regression coefficients. However, this is highly rigid as the smoothing of the underlying function depends on the proportional hazards assumption, which may not be supported by the data and is often overlooked by the investigators [9]. Essentially, the CPH model was designed to measure the effects of covariates on the changing hazard function and not to model patient survival. A flexible family of functions which allows for parametrically modeling the baseline survival function is more appropriate, especially if the proportional hazards assumption is violated in the CPH [12]. The baseline survival has for the most part been ignored because it is left undefined in the CPH model.

In this manuscript we compare CPH with an alternative method of estimating survival in the form of the class of flexible Royston-Parmar (RP) parametric functions [12]. We use the Palliative Performance Scale (PPS) [13] from a cohort of hospice patients. Results from systematic reviews have shown that the patient PPS score is an accurate measure of patient survival in the palliative setting [7], [11]. Furthermore, PPS and CPH model have been used to construct meaningful hospice patient survival estimates in the form of a life expectancy table and survival nomogram [14] and to validate prognosticating scales for hospice patient survival [15], [16], [17].

In addition to PPS, other risk factors such as age, cancer status and gender have been reported to be significant predictors of palliative patient survival in several studies [11], [14]._ENREF_18 In our study we did not adjust for other risk factors because though they may be significant predictors of survival for the cohort of patients in our dataset, they may not be in other palliative settings. Our goal was to demonstrate that the RP family of parametric functions allowed for a direct and flexible modeling of the baseline survival and that it might be formulated so that the impact of the proportional hazard assumption is minimized. We determined if the overall performance and discriminatory ability of RP family of parametric functions is superior to CPH in the sample by using models that were derived and tested on the whole dataset (naïve validation) and using (internal) cross-validation. It is important to note that the RP parametric functions have not been applied to prognostic models in the hospice and palliative settings. It is also important to note that we did not perform external validation, which entailed using a different data set than the one used to create the model[3]. In the next section we briefly discuss PPS, introduce the statistical models and measures of model performance.

## Methods

### Study sample and palliative performance

The patient data were obtained from the Lifepath Hospice and Palliative Care Center licensed since 1983 to serve in Hillsborough County, Florida. Hospice care focuses on pain control and symptom management. To avoid selection bias, we retrospectively and sequentially extracted data for 590 patients who, as of January 2009 were deceased. This study was a retrospective review of the deceased patients' medical records. Only data pertaining to outcomes were collected; personal information was not collected and the data were de-identified prior to analysis. Since we did not collect any information that can identify deceased patients or their family members, under HIPPA rules and regulations (45 CFR 164.512) the requirement for consent does not apply. The study and consent procedures were approved by the University of South Florida Institutional Review Board prior to study initiation. Two research assistants extracted all data necessary to populate the model variables and two faculty members randomly checked 25% of the data for accuracy. The models were tested against observed survival duration.

The Palliative Performance Scale (PPS) was developed and reported by Anderson et al. [13] as a measure of palliative patients' functional status. The scale has 11 possible mutually exclusive levels, which are based on five domains: six levels of ambulation, six levels of activity and evidence of disease, five levels of self-care, five levels of food intake and four levels of consciousness. The scale ranges from PPS of 0% (deceased patient) to PPS of 100% (ambulatory and healthy patient). Numerous studies have studied its prognostic accuracy of survival in a variety of settings and found it provides meaningful estimates of patient survival [10], [14], [15], [18], [19], [20], [21], [22], [23]. PPS has been found to be both valid and reliable [24].

### Model selection and validation

Validating a prognostic model is generally accepted to mean that given a patient population it works in a data set other than the one it is applied to[2], [25]. In other words, the model needs to be tested using a different data set than the one used to create the model[3]. It is also generally accepted that the validation process should follow guidelines and that un-validated prognostic models should not be applied in clinical practice [3], [4], [26]. When validating a prognostic survival model in the regression framework, most attention has been on the value of the prognostic index based on covariates, while the role of the baseline survival function has been largely ignored.

The role of the baseline survival is significant as it quantifies the absolute patient survival probabilities over time. For a vector of covariates **x** and parameter vector **β**, the survival function S(t; **x**) at time t for the CPH model is commonly expressed as 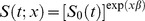, where S_0_ (t) is the baseline survival function, i.e. survival function when all the covariates **x** are equal to zero. In the CPH framework, the estimation of the prognostic index **xβ** does not require the formulation of the baseline cumulative survival function S_0_ (t), which itself can be estimated conditional on the covariate estimates. The two popular methods for estimating baseline survival S_0_ (t) are the Breslow and Kalbfleisch-Prentice methods [27]. Both give similar results in practice, but can lead to “choppy” estimates of the baseline function and are dependent on the proportional hazards assumption.

When the goal of a survival analysis is to estimate hazard ratios (the effect of covariates on the changing hazard function), the baseline function is of no consequence. The CPH is appropriate as the baseline function gets absorbed when coefficient **β**s are estimates by the method of partial log likelihood. However, when the goal is to prognosticate patient survival, there is a need for more flexibility in modeling the baseline survival.

An alternative to the CPH is the RP family of models that resembles the generalized linear models and can be viewed as a parametric extension Cox proportional hazard models [12]. The models are framed to rely on the transformation g(.), such that 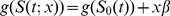. The transformation g(.) can be either from the proportional hazard, proportional odds, Aranda-Ordaz or probit families [12]. We did not consider the Aranda-Ordaz family in this study due to possible interpretational difficulties [12]. Under the proportional hazard link function, the hazard ratio estimates are nearly identical to those estimated under CPH. The attractive feature of the RP baseline survival function is that its shape is preserved, but the location of the baseline distribution function can vary, which allows for flexible model recalibration. Also, the estimate 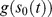 is implemented on log-time scale. It is generally gently curved and smooth, making survival estimates more accurate.

In the RP framework, if the proportional hazard assumption is violated, the probit-link function g(s) = −Φ^−1^(s) can be applied, where Φ^−1^(.) is the inverse standard normal distribution function. The baseline survival function 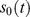 is approximated and smoothed by a restricted cubic spline function with m interior knots. Splines are piecewise polynomials that ensure the overall curve is smooth (see Royston and Parmar [12] for details). Spline-based survival models such as RP have been empirically shown to be superior when the proportional hazard assumption is violated [28]. The optimal number of knots and the comparison among different RP models can be found using the minimum combination of Akaike Information Criterion (AIC), Bayes Information Criterion (BIC) and explained variation statistic R^2^ [29], [30]. The AIC is defined in the usual manner as - 2Log(likelihood) + 2(No. of model parameters), while BIC equals - 2Log(likelihood) + (No. of model parameters)*Log(n). In survival analysis n is interpreted as the number of events rather than the number of patients. The placement of knots in spline modeling is an issue. We have placed the knots at equally spaced centiles of the log-survival times, following published recommendations [31]. For example, for m = 1 the knot is at the 50^th^ centile, for m = 2 the knots are at the 33^th^ and 67^th^ centiles, etc.

We compared RP and CPH by performing internal validation (assessing validity in the population where the development data originated from) on the whole data set (naïve) and using split-sample cross-validation. We performed 10-fold cross-validation by splitting the data into development and validation sets and repeating the process 20 times. The methods can be readily implemented in Stata [32], [33] statistical software using the *stpm* [29] and *stpm2* [34] commands, or in open source statistical software R as *flexsurv* package [35].

### Assessment of model performance

Model performance is the ability of the estimated risk score to predict survival and is assessed using the measures of explained variation, calibration, and discrimination. Calibration refers to how closely the predicted survival at a pre-specified time agrees with the observed survival. For cross-validation, we compared the average fitted probabilities of survival under RP and CPH for the first 15 days to observed probabilities estimated non-parametrically using Kaplan-Meier curves [36].

The Brier score is a quadratic scoring rule that calculates the differences between the actual outcomes and predicted probabilities[37]. Given the predicted probability of survival p_i_ at time t for patient i, and Y_i_ binary (0–1, dead-alive) variable, the Brier score is defined as 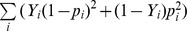. A Brier score of 0 indicates a perfect model, while 0.25 indicates a non-informative model (the value achieved when issuing a predicted probability of 50% to each patient). The Brier score may be scaled by its maximum Brier_max_ = (1 – mean(p_i_)) mean(p_i_) to obtain 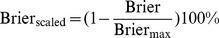. The scaled Brier scores range from 0% to 100% and have interpretation similar to the Pearson correlation coefficient[38].

For a particular risk score, discrimination is the ability to differentiate between the patients who died versus those who survived. The Kaplan-Meier plot of survival for patients in different risk groups can be used to test for separation, indicating that the different risk groups are well defined [39]. For a statistical model, the global measure of the model's discriminatory power is the explained variation statistic R^2^, which measures the variation explained by the fitted model [40]. Higher values of R^2^ indicate greater discrimination. In this study we implement R^2^ for survival models, as described by Royston and Sauerbrei,[41].

The discrimination or Yates slope is a measure of how well the subjects with and without the outcome are separated. It is defined as the absolute difference in mean predictions of survival (mean[p_i_]) between those who died and those who survived at time t[2]. The scaled Brier scores and discrimination slopes were calculated separately for the (naïve) model using the whole dataset and the model derived using cross-validation for t = 1, 2… 100 days. Higher scaled Brier scores and discrimination slopes represent better model performance.

All statistical calculation were performed using Stata version 11.2 [32], [33].

## Results

### Description of the data source

The patient characteristics of the retrospective cohort are summarized in Table 1. The cohort consisted of 293 males (49.7%) and 295 females (50.0%), and 2 (0.3%) with unknown gender. The data were collected starting from patients' entry into hospice care until death for all 590 patients. The mean, median and range of survival times for the patients by PPS at admission, age, gender, cancer status, and diagnosis category are given in Table 2. The table shows that the median survival was fairly evenly distributed across age groups and gender, but unevenly across the cancer status and initial diagnosis category. All patients were assigned PPS at the time of admission to hospice care. Since PPS score of 0% means that the patient is dead, the data were transformed so that the PPS score of 10% was set as the baseline. There were only 15 total observations for PPS = 60%, 70%, 80%, so they were combined with PPS = 50% to obtain meaningful survival estimates. Fourteen patients had missing values for PPS.

**Table 1:** Patient characteristics.

Variable	Result
Total no. of patients	590 (100%)
Age at Treatment	
<45	37 (6.3%)
45–64	187 (31.7%)
65–74	110 (18.6%)
75–84	129 (21.9%)
85+	127 (21.5%)
Gender	
Male	293 (49.7%)
Female	295 (50%)
Unknown	2 (0.3%)
No. of patients with cancer/noncancer	
Noncancer	363 (61.5%)
Cancer	227 (38.5%)
Diagnosis category for cancer	
Brain	10 (1.7%)
Gastrointestinal	35 (5.9%)
Genital-female	12 (2%)
Genital-male	12 (2%)
Head and neck	8 (1.4%)
Hematopoietic	10 (1.7%)
Pancreas	24 (4.2%)
Respiratory	49 (8.3%)
Skin	2 (0.3%)
Urinary	4 (0.6%)
Other	61 (10.3%)
Diagnosis category for noncancer	
AIDS	12 (2%)
Cardiovascular	74 (12.5%)
Neurological	119 (20.2%)
Respiratory	37 (6.3%)
Other	121 (20.6%)

**Table 2:** Survival time by age, gender, diagnosis and initial PPS.

	Survival Times (in Days)			
Variable	Mean (95% CI)	Median (95% CI)	Range	No. of Patients (%)
Total no. of patients				
Overall	14 (12, 17)	6 (5, 6)	1–371	590
Age at Treatment				
<45	15 (8, 22)	8 (4,12)	1–95	37 (6.3%)
45–64	14 (11, 17)	7 (5, 9)	1–114	187 (31.7%)
65–74	14 (8, 20)	5 (4, 6)	1–271	110 (18.6%)
75–84	14 (8, 20)	6 (5, 7)	1–371	129 (21.9%)
85+	15 (9, 21)	5 (4, 6)	1–313	127 (21.5%)
Gender				
Male	14 (10, 18)	6 (5, 7)	1–371	293 (49.7%)
Female	15 (11, 19)	6 (5, 7)	1–271	295 (50%)
No. of patients with cancer				
Noncancer	12 (8, 16)	5 (4, 6)	1–371	363 (61.5%)
Cancer	17 (14, 20)	9 (7, 11)	1–113	227 (38.5%)
Diagnosis category for cancer				
Brain	27 (16, 39)	28 (14, 42)	3–55	10 (1.7%)
Gastrointestinal	21 (14, 29)	11 (5,17)	1–82	35 (5.9%)
Genital-female	15 (6, 24)	8 (1, 15)	2–55	12 (2%)
Genital-male	26 (7, 45)	13 (4, 22)	1–100	12 (2%)
Head and neck	10 (2, 18)	5 (1, 9)	1–36	8 (1.4%)
Hematopoietic	4 (2, 6)	3 (1, 5)	1–10	10 (1.7%)
Pancreas	18 (7, 29)	7 (3, 11)	1–113	24 (4.2%)
Respiratory	15 (10, 20)	10 (7, 13)	1–71	49 (8.3%)
Skin	11	11	11–11	2 (0.3%)
Urinary	25 (1, 58)	9 (1, 39)	4–76	4 (0.6%)
Other	17 (12, 22)	9 (5, 12)	1–103	61 (10.3%)
Diagnosis category for noncancer				
AIDS	18 (3, 33)	8 (1, 15)	1–85	12 (2%)
Cardiovascular	14 (5, 23)	5 (3, 7)	1–271	74 (12.5%)
Neurological	8 (5, 11)	5 (4,6)	1–77	119 (20.2%)
Respiratory	25 (1, 49)	3 (1, 5)	1–371	37 (6.3%)
Other	11 (1, 15)	5 (4, 6)	1–174	121 (20.6%)
Initial PPS Score				
PPS 10%	5 (3, 7)	3 (2, 4)	1–77	188 (32.6%)
PPS 20%	16 (8, 24)	5 (4, 6)	1–371	125 (21.7%)
PPS 30%	15 (11, 19)	7 (5, 9)	1–140	123 (21.4%)
PPS 40%	24 (18, 30)	14 (11, 17)	1–147	96 (16.7%)
PPS 50–80%	28 (21, 35)	18 (9, 27)	1–76	44 (7.6%)

**Figure 1:**
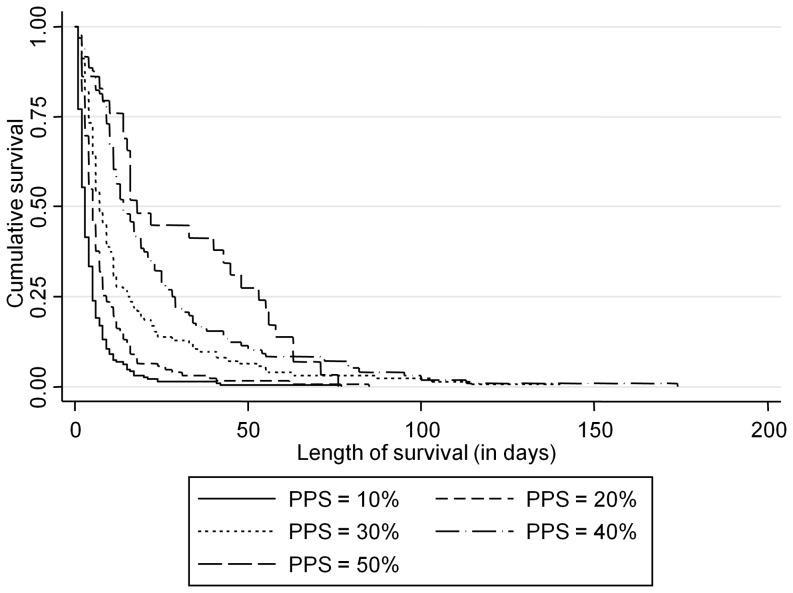
Kaplan-Meier survival curves by initial PPS.

**Figure 2:**
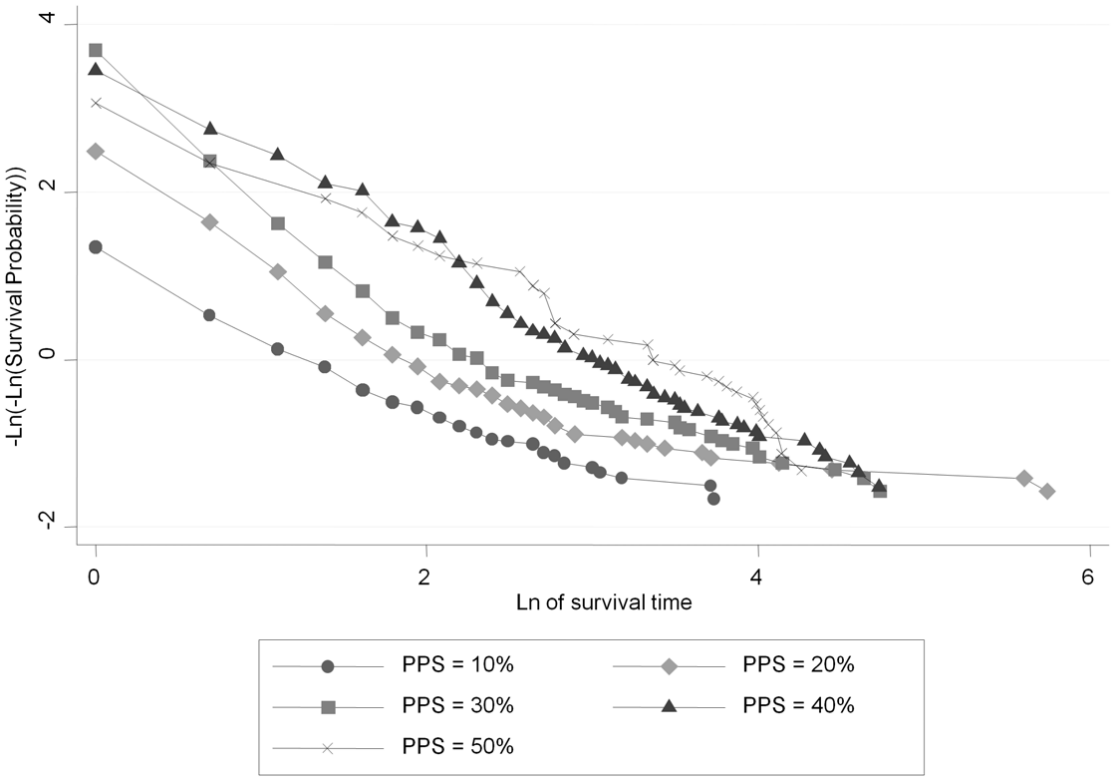
Test of the proportional hazards assumption under CPH for initial PPS.

The time of admission was the starting point for survival time. The Kaplan-Meier curves stratified by initial PPS level are shown in Figure 1. The curves show good separation indicating that the different risk groups are well defined. The log-rank test for equality of survival curves was highly significant at P = 0.001. The global test based on Schoenfeld residuals showed that the proportional hazard assumption was violated for PPS (P-value <0.001), which can also be seen from the un-parallel natural log-plot of survival curves (Figure 2).

**Figure 3:**
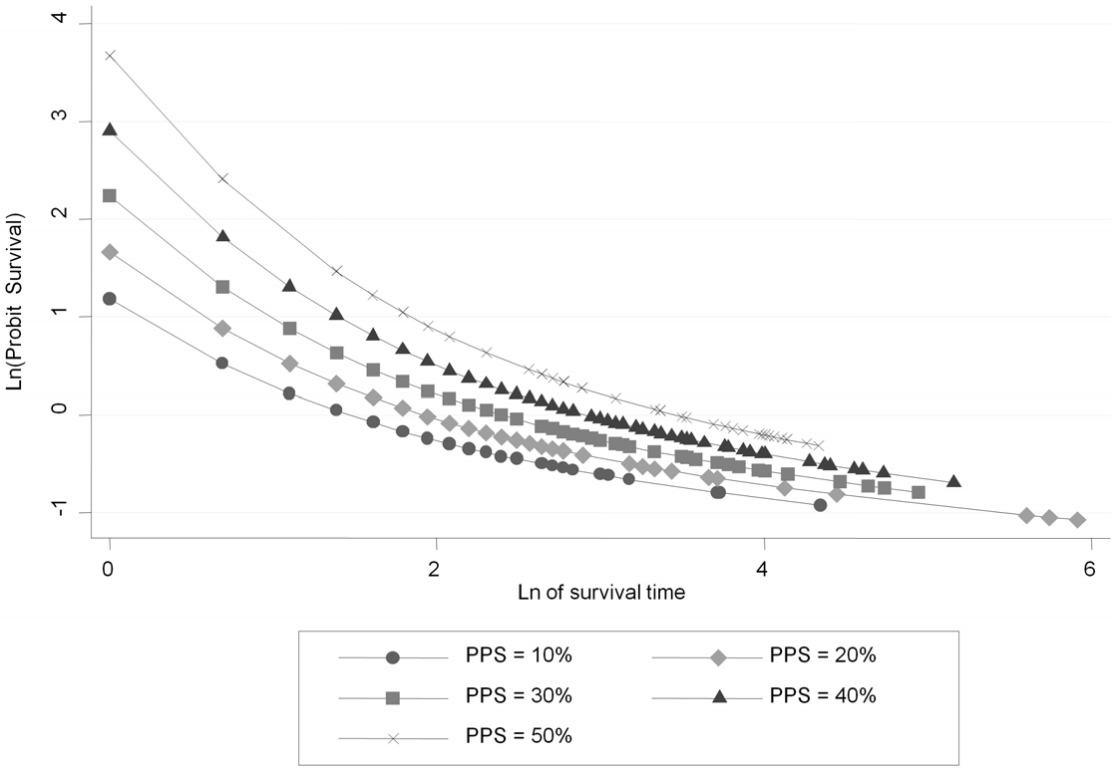
Test of the probit assumption under RP for initial PPS.

Table 3 lists AIC, BIC and R^2^ values for 5 knots under the proportional hazard, proportional odds and probit RP families; the minimum combination in each is underlined. The number of optimal knots was found to be m = 1 under the probit model. The improvement in fit with the probit model can be seen from the parallel survival curves of log-probit against natural log time (Figure 3).

**Table 3:** Criteria for the choice of scale in the RP model**.**

No. of knots m	PH	PO	Probit
	AIC, BIC, R^2^	AIC, BIC, R^2^	AIC, BIC, R^2^
0	2033, 2042, 0.156	1887, 1896, 0.321	1872, 1881, 0.295
1	1889, 1902, 0.178	1883, 1896, 0.322	1858, 1871, 0.298
2	1871, 1888, 0.170	1870, 1887, 0.312	1857, 1874, 0.296
3	1870, 1892, 0.172	1870, 1892, 0.311	1858, 1880, 0.297
4	1865, 1892, 0.171	1865, 1891, 0.310	1855, 1881, 0.296
5	1866, 1896, 0.171	1865, 1896, 0.309	1856, 1886, 0.296

**Figure 4:**
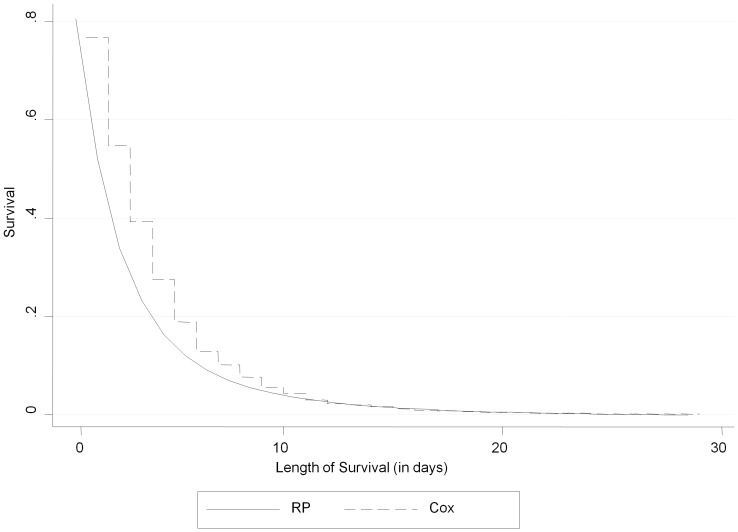
Baseline survival functions under CPH and RP models.

R^2^ was higher in the RP model (R^2^ = 0.298; 95% CI: 0.236–0.358) than the Cox model (R^2^ = 0.156; 95% CI: 0.111–0.203), indicating that the RP model explained significantly more variation than CPH. To illustrate the differences for the baseline function, Figure 4 shows plots of the CPH and RP baseline survival functions. The CPH baseline survival is “choppy” to approximately day 12, while the RP is smooth. The two baseline functions converged at around day 12.

**Figure 5:**
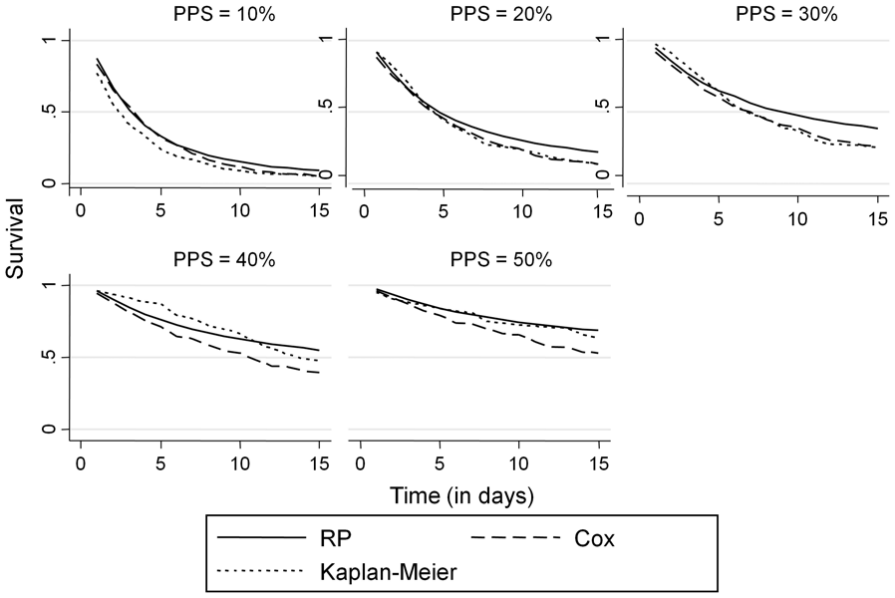
Predicted survival by PPS under RP and CPH compared with the Kaplan-Meier estimates in the validation data.

**Figure 6:**
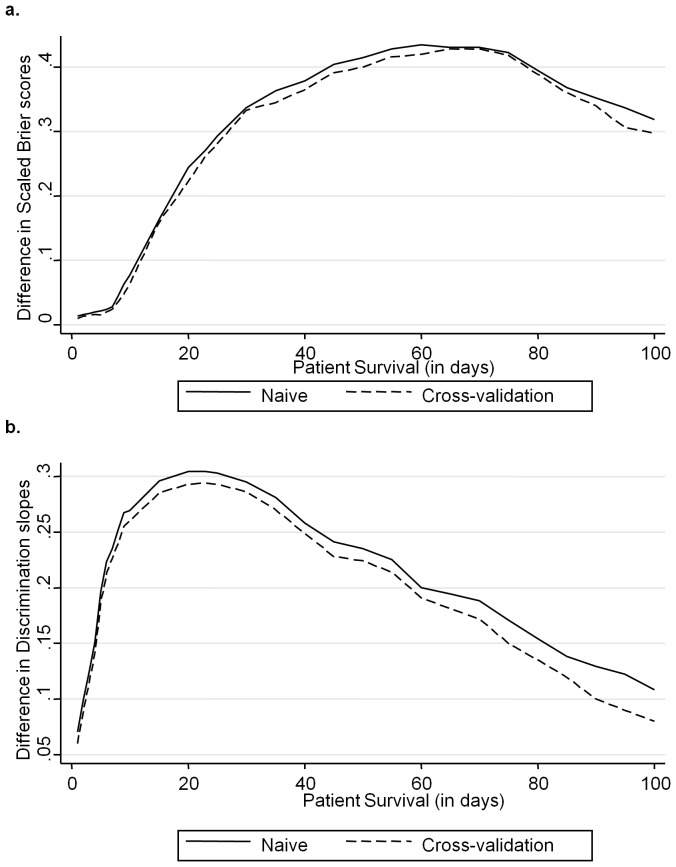
Difference between Brier scores for RP and CPH models (6a) and between discrimination slopes for RP and CPH models (6b) as a function of patient survival times in the naïve (whole) data set and cross-validated data set. Both are consistently higher for RP indicating better accuracy and discrimination.

Cross-validation showed that the relation between the two predicted survival estimates is approximately linear, with RP model consistently estimating a higher probability, which is particularly evident for higher scores of PPS corresponding to longer survival times (Figure 5). Overall, the predicted probabilities under RP tended to be closer to the Kaplan-Meier estimates than CPH. The plot of the consistently positive differences between RP and CPH scaled Brier scores (Figure 6a) and discrimination slopes (Figure 6b) showed that the RP model discriminated better across patient survival times for both the full (naïve) and cross-validated models. This suggested that the higher value of R^2^ under RP was not due to over-fitting.

## Discussion

The results from our study show that RP family of models predicts survival more accurately than CPH through its flexible modeling of the baseline survival function. Using the RP flexible baseline function modeling would allow for more precise calibration in the prognostication phase than CPH. As Figure 5 illustrates, the predicted RP survival probabilities are consistently higher for higher values of PPS, and closer to the Kaplan-Meier estimates of survival. We suspect that both the robust modeling of baseline survival and overall model fit provide for better survival estimation.

There are limitations to our study, the primary one being the use of retrospective data. The RP family of parametric functions needs to be applied prospectively to assess accuracy of prognostic models through external validation. Furthermore, the dataset was limited to the hospice setting with no censored observations and with majority of patients having a very short follow-up time. For future studies, application of the proposed methodology should account for these limitations, and comparisons with parametric prognostic survival models should be explored.

The flexible models discussed in this paper could greatly improve the ability of researchers to accurately predict survival. An advantage of RP is that it can be used to validate published models for which the original individual patient data are unavailable. If the scale used (hazard, probit or odds), the knot positions, and the estimates of prognostic indices are known, then it would be possible to use RP. In the case of CPH this is not possible, since the baseline function would not be available.
